# Modeling Leukocyte-Leukocyte Non-Contact Interactions in a Lymph Node

**DOI:** 10.1371/journal.pone.0076756

**Published:** 2013-10-28

**Authors:** Nicola Gritti, Michele Caccia, Laura Sironi, Maddalena Collini, Laura D'Alfonso, Francesca Granucci, Ivan Zanoni, Giuseppe Chirico

**Affiliations:** 1 Dipartimento di Fisica, Università degli studi di Milano-Bicocca, Milano, Italy; 2 Dipartimento di Biotecnologie e Bioscienze, Università degli studi di Milano-Bicocca, Milano, Italy; 3 Dynamics of Immune Responses, Division of Immunology, Transplantation and Infectious Diseases, San Raffaele Scientific Institute, Milano, Italy; Uniform Services University of the Health Sciences, United States of America

## Abstract

The interaction among leukocytes is at the basis of the innate and adaptive immune-response and it is largely ascribed to direct cell-cell contacts. However, the exchange of a number of chemical stimuli (chemokines) allows also non-contact interaction during the immunological response. We want here to evaluate the extent of the effect of the non-contact interactions on the observed leukocyte-leukocyte kinematics and their interaction duration. To this aim we adopt a simplified mean field description inspired by the Keller-Segel chemotaxis model, of which we report an analytical solution suited for slowly varying sources of chemokines. Since our focus is on the non-contact interactions, leukocyte-leukocyte contact interactions are simulated only by means of a space dependent friction coefficient of the cells. The analytical solution of the Keller-Segel model is then taken as the basis of numerical simulations of interactions between leukocytes and their duration. The mean field interaction force that we derive has a time-space separable form and depends on the chemotaxis sensitivity parameter as well as on the chemokines diffusion coefficient and their degradation rate. All these parameters affect the distribution of the interaction durations. We draw a successful qualitative comparison between simulated data and sets of experimental data for DC-NK cells interaction duration and other kinematic parameters. Remarkably, the predicted percentage of the leukocyte-leukocyte interactions falls in the experimental range and depends (≅25% increase) upon the chemotactic parameter indicating a non-negligible direct effect of the non-contact interaction on the leukocyte interactions.

## Introduction

The Immune System defends our organism from pathogens via innate and adaptive immune responses that are triggered by a cascade of interactions between different leukocytes [1]. One of the most known cell-cell interactions involves mature Dendritic Cells (DCs) and T cells leading to the activation of adaptive immunity. Recent reports showed clear evidence that DCs play a major role also in the activation of Natural Killer (NK) cells. This process involves direct DC-NK cell interactions [2]–[4] and release of a number of cytokines [5].

The interactions between leukocytes have been visualized with a number of imaging techniques [6]. Particularly effective to this aim are recent Two-Photon Microscopy (TPM) studies. This approach allows to quantify the cell diffusion coefficients, the statistics of the cell motion [7] and the duration and distribution of cell-cell interaction times [8]–[13]. The sensitivity and accuracy of the algorithms employed to reveal the interactions between the leukocytes have been specifically questioned recently [7]. The two-photon in-vivo microscopy experiments provide a wide range of parameters that characterize the cell diffusion and the cell-cell interaction. However, several questions can be raised regarding the operative definition of leukocyte-leukocyte interaction. The detection of an interaction can be affected by methods used for the image analysis. Often the detection of the interaction between leukocytes is made by visual inspection of the acquired images, and this may introduce unwanted bias to the data. Even when a quantitative algorithm is employed [7], it is difficult to reduce the complexity of the motion to a test of few selected kinematic parameters. Additional problems arise from the limited observation time window and by the loss of tracking due to poor signal/noise in the images [7].

These issues could be addressed with the help of numerical simulations that should take into account a variety of processes. In particular our thesis here is that leukocyte kinematics in vivo is affected by membrane receptors mediated direct contacts, but also by leukocyte-leukocyte signaling producing effective non-contact interactions. Signaling among cells have been addressed in the literature [14]–[16], but these studies were not widely applied to in vivo two-photon microscopy data analysis. Intermittent directional motion of the leukocytes [13] observed in-vivo are an indication of non-contact interactions among leukocytes. These interactions have also alternative sources, for example direct interaction with the tissue in their motion, in addition to chemo-attraction mediated by chemokines [17], [18] or chemokinesis [15]. The outlined scenario is complex and the role played by non-contact interactions is not easy to be discerned. We are not looking here for a comprehensive model that describes such complex scenario and would require further developments that take advantage of the sophisticated models reported in the literature [19]–[24]. We address instead the extent of the effect of non-contact interactions on the observed kinematics of the leukocytes, primarily on the interaction duration. To this purpose we will employ a simplified mean field description of the leukocyte-leukocyte non-contact interactions. Contact interactions will be accounted for in an a-specific way by means of a space dependent leukocytes friction coefficient.

We develop then a simple numerical algorithm that takes into account the leukocyte diffusion and some sort of action at a distance between the leukocytes, to simulate which we are inspired by the general theoretical framework set up by Keller and Segel [25] to treat chemotaxis. We derive a solution of the Keller-Segel model for a simplified expression of the cell-cell interaction potential in the presence of chemokines. This is based on the assumption that the less mobile dendritic cells act as a slowly varying source of chemokines that attract the highly mobile NK cells. We apply this solution to an extensive numerical simulation study with statistics comparable to that typically obtained by two-photon microscopy experiments. Several other elegant applications and solutions of the Keller-Segel model have been reported in the literature [14]–[16], also for the analysis of leukocytes motion. However these works deal with in vitro studies, for example the Millipore filter diffusion experiment [15] or the under-agarose migration assay [26] or micro-organisms observations, such as parasitoids aggregation in response to chemical signaling [14], and in some cases report full simulative developments of the Keller -Segel original model [25].

The relevant parameters of the Keller -Segel model are the chemotaxis sensitivity parameter, χ, the chemokine degradation rate, k and chemokine source density **J** (**r**,t) (see **Eq.9A**, below). The analytical expression of the effective attractive potential can be obtained with the assumption that the chemokine production time is much larger than their diffusion time over a distance of a few cellular diameters. This solution is then employed in numerical simulations of the chemokine mediated interaction between the highly mobile NK cells and the DCs. In this way we can predict several geometrical and dynamical parameters of the leukocytes motion, the probability distribution of the duration of the interactions between NK and dendritic cells and its dependence on the sensitivity parameter. On this basis we can draw qualitative comparison with experimental data.

## Materials and Methods

### Ethic Statement

We declare that all experiments were performed using protocols approved by the University of Milano-Bicocca Animal Care and Use Committee (also in agreement with the European rules, 86/609/EEG and with the International Guiding Principles for Biomedical Research Involving Animals, as developed by the Council Organizations of Medical Sciences and the Guide for the Care and Use of Laboratory Animals; http://ec.europa.eu/environment/chemicals/lab_animals/revision_en.htm). Mice were housed in containment facilities of the animal facility and maintained on a regular 12∶12 hour light:dark cycle with food and water ad libitum.

### Free cell diffusion simulation

Langevin equation comprises an inertial, a dissipative and a stochastic term. If we integrate the Langevin equation over times much larger than the velocity relaxation time, we can get rid of the inertial term. Within these assumptions the description of the Brownian motion of a set of identical particles that move in a fluid at thermal equilibrium can be obtained by the following equations: [27]–[30].
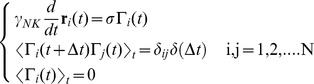
(1)


All symbols used in the equations are listed and defined in **Table 1**. The stochastic forces 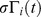 have Gaussian distribution with variance σ. The symbol 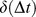 indicates the delta function with argument equal to the integration time 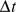. This stems from the Markovian character of the process and accounts for the complete decoupling of successive integrations steps [31].

**Table 1:** Parameters used in the derivation of the NK-DC simulations.

Parameter	Description	Range	Eq.
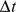	integration time step;	1 s	4
M	Number of simulated steps	10^7^	4
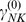	intrinsic friction, Natural Killer cells	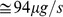	2,5
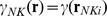	space dependent friction coefficient, i-th NK cell		7
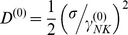	Intrinsic NK cells diffusion coefficient	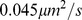	5,10
c (**r**,t)	chemokine concentration		9,12,14
J (**r**,t)	source of chemokines on the dendritic cell		9
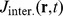	chemokines source on the dendritic cell under interaction		11
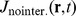	chemokines source on dendritic cell under no interaction		13
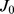	Intrinsic source density on a dendritic cell	10^3^–10^6^ molecules/s	11,13
τ	Interactions' activation/deactivation time	3600s	11,13
D_CK_	chemokines' diffusion coefficient	10 µm^2^/s	9
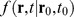	conditional probability for the NK cells		3, 9
R (t) = 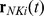	position of the NK cell		8
σ	variance of dr (t)/dt, see Eq.2		1,2,3,10
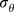	variance of azymuthal angle for worm like chain model	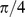	S2
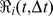	Gaussian distributed variable for NK cell Brownian motion		8
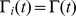	Gaussian distributed variable with zero average and variance = 1		1,6
K	characteristic degradation time of the chemokines	300–900 s	9
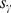	extension of the spatial dependence of the NK friction coefficient	25 μm	7
*k*_γ_	amplitude of the space dependence of the NK friction coefficient	10	7
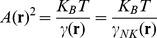	amplitude of the force induced by space dependence of 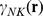		6
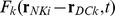	force acted by the k-th dendritic cell on the i-th NK cell at time t		6
?	Chemotactic parameter	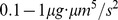	9
N_CK_	Number chemokines produced by a dendritic cell	10^5^	
n_CK_	Estimate of the CK number concentration	8.4–9.6 10^11^ cells/cm^3^	
N_NK_	Number of simulated NK cells	10^2^	4
m_NK_	Mass of a NK cell	800–1000 10^−12^g	
V_DC_	Volume of the DC	≅6000–32000 μm^3^	
r_NK_	Radius of a NK cell	5 μm	
a_0_	Minimum distance for interaction	25 μm	
T_th_	Duration threshold for interactions	300–600s	
T	Bulk temperature	310 K	5
η	Bulk viscosity	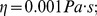	5

List of the parameters used in the derivation of the simulations of the NK and dendritic cells with an indication of the value and the equation in which the parameter was defined and/or used. When no number is given for the equation, the parameter was used for a direct estimate in the text.

The second line of **Eq.1** states that the stochastic forces are uncorrelated on the scale of the velocity relaxation time, 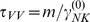, where m and 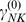 are the mass and the friction coefficient of the NK cell. We compute the cell frictional coefficient, 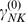, as that of a rigid sphere of radius 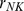
_≅_5 µm, 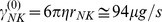, and the diffusion coefficient from the Stokes-Einstein relation, 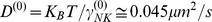. In these relations η, T and K_B_ are the solution viscosity and temperature and the Boltzmann constant. With this choice, the velocity relaxation time is few microseconds. Finally, the force variance is related to the cell translational diffusion coefficient, D ^(0)^, and the friction coefficient as:
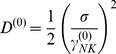
(2)


The Langevin equation (**Eq.1**) corresponds to the Fokker-Planck equation for the conditional probability of the NK cells, 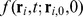:
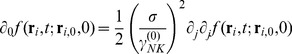
(3)


In **Eq.3** and in the main text we use the notation, 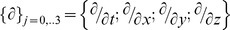, 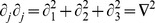 and in general we consider summed the repeated indices. The integration time chosen here, Δt  = 1s, is sufficiently larger than the velocity relaxation time and allows us to integrate the intracellular forces as detailed below. The Gaussian distributed random variable 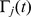, is computed by means of a Box-Mueller algorithm [32].

### Free cell diffusion simulation

The Brownian motion of a set of identical Natural Killer (NK) cells diffusing (diffusion coefficient D ^(0)^) in a fluid at thermal equilibrium was simulated, for integration times Δt much larger than the velocity relaxation time,[31] by means of the following finite differences algorithm [28], [29], [33]:
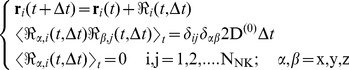
(4)


The above form is the integration of the Langevin equation (**Eq.1**) as in the Euler-Maruyama algorithm and the stochastic variables 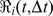 are normally distributed. The diffusivity σ (see **Eq.2**) that appears in this equation is related to the random displacement 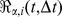. The integration time was assumed Δt  = 1s throughout this study and the simulations included N_NK_  = 100 NK cells in a 500×500×50 µm^3^ box and were run for a total number of steps M = 10^7^ with the following parameters (see also **Table 1**):
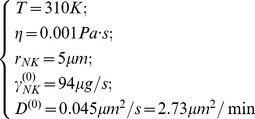
(5)


**Figure 1:**
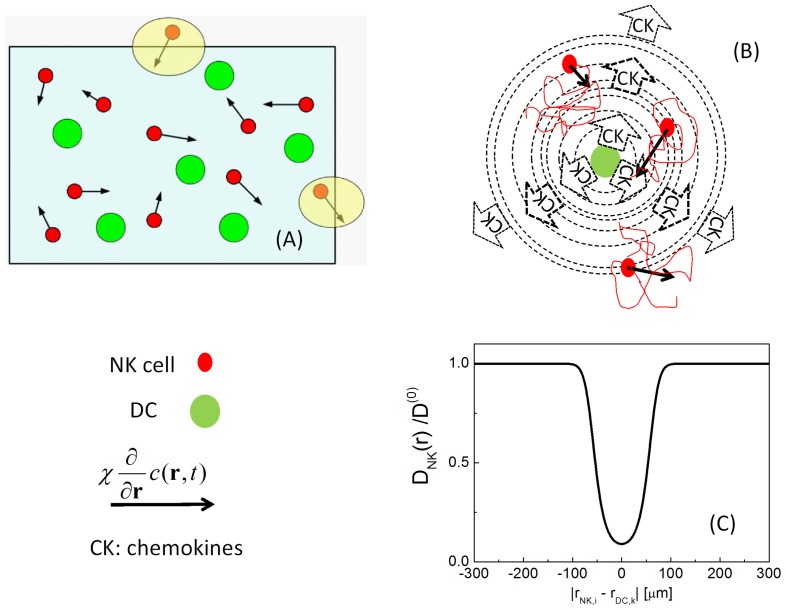
Sketch representing the main features of the simulation algorithm. Panel A: implementation of the boundary conditions. When a NK cell (red dots) exits the simulation volume, another NK cell enters it from a random location. The dendritic cells are represented as fixed green circles and the solid arrow indicate the chemotactic force 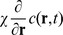. Panel B sketches action at a distance acted by a dendritic cell that is simulated as a source of chemokines whose spread is represented by dashed circles and dotted arrows. The red lines represent the actual movement of NK cells that is largely determined by the Brownian component. Panel C represent the space dependent NK cell diffusion coefficient D_NK_ (**r**) normalized to the intrinsic NK cell diffusion coefficient,
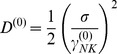
.

The NK cell friction coefficient, 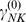, was computed from the cell radius, r_NK_, according to the Stokes-Einstein equation. The Gaussian distributed random displacement, 
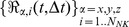
, defined by the first two moments in **Eq.4**, was computed as detailed in File S1 (paragraph S1: “Test of the Random Number generator”). The periodic boundary conditions reproduced the experimental situation in which the number concentration (number of cells per unit volume) of leukocytes in a field of view fluctuates around an average value with a Poisson distribution. For sake of simplicity we kept constant the instantaneous number of NK cells in the simulation box. If one of the NK cells exits from the simulation volume at a specific time step, we assume that this specific NK cell does not re-enter the volume: the track of this specific NK cell will then be limited to this simulation frame. A new NK cell is randomly located close to the box boundary at the next simulation step (**Fig 1A**).

Tests of the numerical integration were run on the free cell diffusion by computing the average mean square displacement, 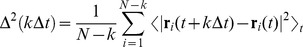 as a function of time. From its slope we obtained the cell diffusion coefficient that resulted always in good agreement (within 1±0.5%) with the input value (see **File S1**, **Fig S1** in paragraph S1: “Test of the Random Number generator”).

### Interacting cells diffusion algorithm

We consider the interaction of a set of mobile NK cells with immobile dendritic cells that act as a center of chemokine production. The situation is modeled by assuming that a source of chemokines, fixed in space and variable in time, acts as chemo-attractant for the NK cells. This action is represented by a deterministic force that depends on the distance between the dendritic and the NK cells and the time: 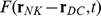. We need to take into account also other major features of the leukocyte dynamics in our simulations: their interaction with the lymph node tissue and the contact antigen recognition that occurs between NK cells and dendritic cells. We assume that the overall effect of these interactions can be captured by slowing down the NK motion while approaching the dendritic cells and simulated via a space dependent friction coefficient (see **Eq. 7**), 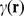, whose spatial extension is ≈10–15 µm (s_γ_ in **Eq. 7**). The finite difference Langevin equation (Δt >> τ_VV_, the velocity correlation time, see Materials and Methods) becomes then [28], [29] (**Fig 1B**):
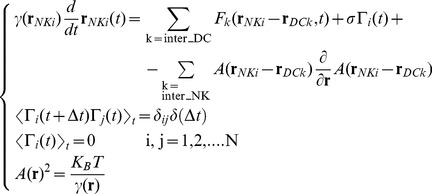
(6)


The sum in **Eq. 6** runs over all the dendritic cells that interact with the selected (i-th) NK cell. The position of the i-th NK cell and of the k-th dendritic cell are 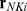 and 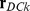, respectively. The term 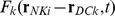 in **Eq.6** is the force acting on the i-th NK cell due to its interaction with the k-th dendritic cell. The friction coefficient of the NK cells, 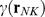, in the proximity of the dendritic cells is described by a Gaussian function of the type (**Fig 1C**):
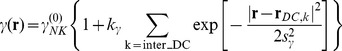
(7)


This friction coefficient corresponds to a reduced diffusion coefficient, 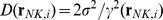 in the proximity of the dendritic cell, and the space dependent term in the Langevin equation (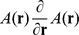 in **Eq.6**) can be assimilated to an attractive force. The range of the effect of the contact interactions is set to 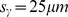 from the centroid of the dendritic cells (according to microscopy images of the dendritic cells [1]) and the amplitude to 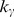  = 10 (see **Fig 1D**). The space dependence of the friction coefficient induces a slight slowing down of the NK cell in proximity of the attracting dendritic cell where the interactions mediated by chemical signals are most effective (see below section, *Metropolis algorithm for interactions*). The specific value of the amplitude k_γ_, in the range 1≤ k_γ_ ≤20, does not markedly affect the result of the simulations discussed in the Results.

The integration of **Eq. 6** was performed by sampling three Gaussian stochastic variables, 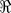
_x_ (t), 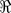
_y_ (t), 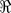
_z_ (t), according to [28], [29], [33]:
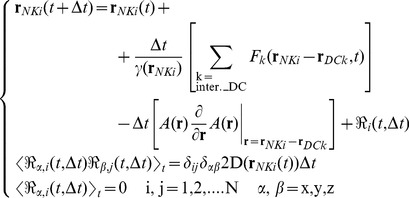
(8)


In our model the interaction forces 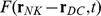 are due to the chemotactic action of the chemokines emitted by the dendritic cells. We will describe such a contribution within the framework of the Keller-Segel model of chemotaxis (see Results) [25].

### Two-photon microscopy

A direct optical microscope (BX51, Olympus) was used to follow the interactions between dendritic and NK cells in lymph nodes. The infrared laser source (Mai Tai HP+DeepSee, Spectra Physics, USA; with pulses of 120 fs full width at half maximum and 80 MHz repetition frequency, DeepSee pulse width compensator) is coupled to the microscope through the FV300 (Olympus, Japan) scanning head. All the images were acquired under two-photon excitation at 800 nm through a 20 X, 0.95 NA, 2 mm WD, Olympus objective (XLUMPlan FI, Olympus, Japan). The fluorescence signal is directed to a non-descanned unit and split into three channels (blue, green and red channel) by two dichroic beam splitters [34]. The acquisition is performed through the Olympus Fluoview acquisition program with an extension to a three channel board (Olympus, Japan). Additional details on the setup and its optical characterization can be found in [34].

The leukocyte interactions data were acquired on explanted lymph nodes. All mice were sacrified via cervical dislocation. The animals from which we explanted lymph nodes were CD11cDTR mice on BALB/c background [35]. These mice express GFP under the control of the CD11c promoter, a dendritic cell specific marker. Therefore GFP is expressed exclusively by dendritic cells. Lymph nodes were analyzed ex-vivo by keeping them in complete IMDM medium (IMDM-10 complete medium: IMDM, 10% heat-inactivated FBS (EuroClone), 2 mM L-glutamine, 100 U/ml penicillin, 100 μg/ml streptomycin, 50 μM 2-mercaptoethanol (Sigma-Aldrich)) saturated with oxygen [34].

NK cells were purified from RBC-lysed splenocytes by MACS positive selection using CD49b (DX5), microbeads (Miltenyi Biotec, D). Purity was assessed by FACS and was routinely between 93% and 96%. Cells were labeled with 1 µM CMPTX (Invitrogen, NL), according to Invitrogen recommendations. NK after labeling with CMPTX were injected i.v. (tail vein), 10 millions per mouse, 24 hs before the experiment. The fluorescently labeled cells were found in the popliteal and brachial lymph nodes about 24 hours after injection. Their localization persisted for at least 72 hours. In order to stimulate the dendritic cells we employed lipopolysaccharide (LPS). This compound was injected sub cute in the mouse footpad, 1 µg per mouse.

For microscopy experiments on explanted lymph nodes, the entire microscope is surrounded by a custom made thermostatic cabinet in which the temperature is kept at 37°C (air thermostating by “The Cube”, Life Imaging Services, Basel, CH) and physiological conditions are guaranteed during the experiments by flowing 37°C buffer solutions saturated with a mixture of 95% O_2_–5% CO_2_ in the lymph node chamber on the objective plane. The lymph-nodes were oriented suitably in order to decrease optical depth due to undesired fat layers and kept in position by a small drop of agarose gel [34].

Several tests were performed to validate the ex-vivo analysis of the data. The motility and distribution of NK cells within the lymph nodes were evaluated on 2-photon microscopy images and movies both intravitally and in explanted lymph nodes [34], [36]. In order to set up the ex-vivo microscopy analysis, imaging was first performed intravital in live animals and ex vivo on explanted lymph nodes [34], [36], [37]. The endogenous and exogenous NK cells were found mainly at the border of the T cell area in both intravital analyses and explanted lymph nodes (in agreement with [12]). Non-activated NK cells were highly motile in steady-state conditions and the mean 3-dimensional (3D) velocities of transferred NK cells, as measured in vivo and ex vivo experiments, agreed with data available for endogenous NK cells [12]). Finally T cells showed an average instantaneous speed of 12±2 µm/min [37] in agreement with available data [11].

Volocity (Perkin-Elmer Inc.) was used to analyze recorded movies. The extracted traces were then analyzed for the measure of the interaction time by means of a specifically designed MatLab (MathWorks Inc.) code.

The simulation code developed here and used to run the simulations reported in the results section can be downloaded from http://moby.mib.infn.it/~chirico/.

## Results

The simulation of the NK motion around dendritic cells was developed within the simplified framework described in the Introduction. We started from freely diffusing NK cell. Then we implement the interactions of these cells with the tissue by means of a Worm like chain method [38]. Finally we implement the interaction field between the NK cells and the dendritic cells, which is derived from the Keller-Segel model [25]. This model was developed in the ′70s to describe the chemotaxis of bacteria particularly in the process of the slime molds aggregation [39]. Keller and Segel takes the cellular motion essentially random with a flux that is composed of two terms. The first term corresponds to a chemotactic flux that in analogy to the Fourier's law of cooling is proportional to the gradient of the chemo-attractant. The second term is the random diffusion of the cells. The equation is closed by providing a diffusion equation for the chemo-attractant with a degradation term.

**Figure 2:**
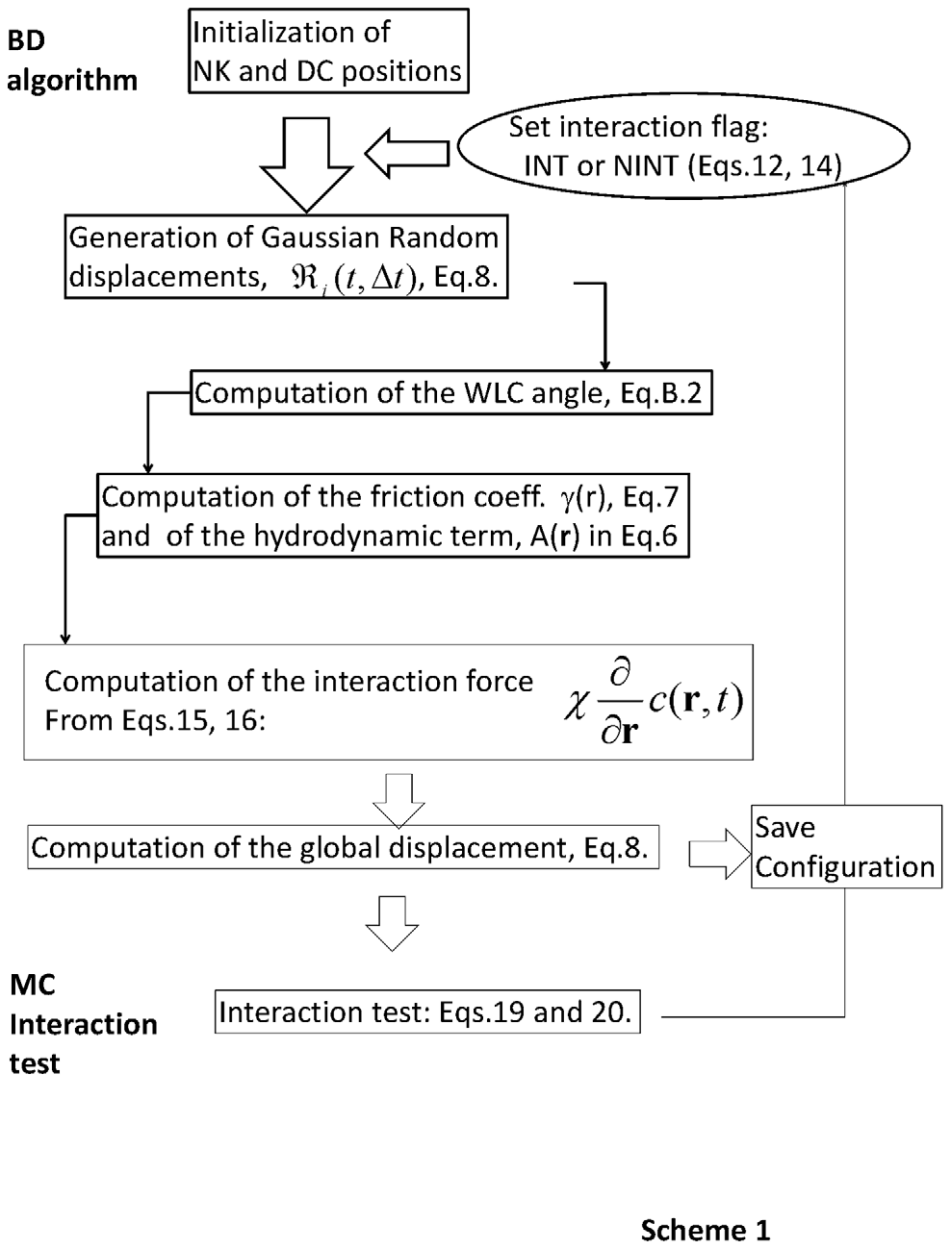
Flowchart of the mixed Monte Carlo-Brownian Dynamics algorithm for the simulation of the leukocyte-leukocyte non contact interactions.

The parameters employed in this derivation and their range are listed in **Table 1** and a sketch of the major features of the algorithm used is reported in **Fig 1B** and in **Fig 2**.

### Modeling of the interactions between NK and dendritic cells

The modeling of a lymph node (LN), that is already a very complex system, or even the whole immune system, has been addressed in the literature with elegant methods [19]–[24]. This is not our aim here. We want instead to estimate the effect of non-contact interactions among leukocytes under the action of an external stimulus that simulates the presence of a pathogen. We will assume a well studied model to describe non contact interactions, the Keller-Segel model [25] of chemotaxis. This choice must be taken more than an inspiration and a mathematical tool than the actual description of the variety of possible leukocyte-leukocyte interactions acting at a distance.

**Figure 3:**
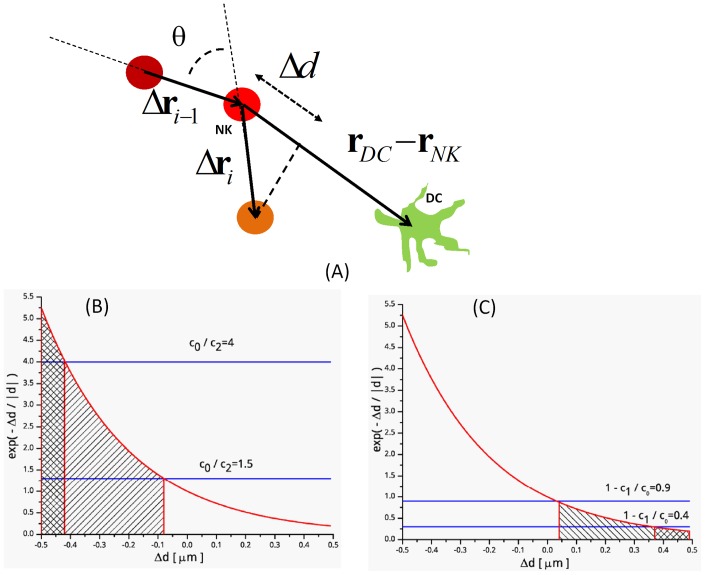
Sketch of the NK cell motion. The NK cell is sketched as a red-orange circle: the brightness of the red color codes for increasing simulation time. Panel A: 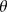 is the deviation angle sampled to implement for the Worm Like Chain model for the diffusion of the NK cell in the tissue. Monte Carlo algorithm for the cell-cell interactions: definition of the displacement parameter, Δd, used for the Metropolis test for the interaction onset. The symbols in panels B and C are: 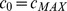, 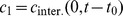
and 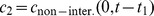. Panel B reports an example of the *interaction_start* test. The root mean square displacement is 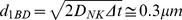. Depicted are two cases corresponding to 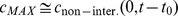 and 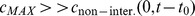. In the first case **Eq. 17** is satisfied even if 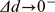, in the second case **Eq. 17** is satisfied only if 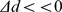. Panel C reports an example of the *interaction_stop* test. In this case when 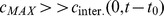 **Eq.18** is satisfied even if 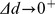 while when 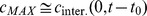 **Eq.18** is satisfied only if 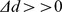.

However, we first need to develop a minimal model that describes the major characteristics of leukocyte dynamics even in the absence of the stimulus. As discussed in File S1 (S2: *Un-stimulated lymphocyte stochastic motion within a lymph node”* in **File S1**) a model that qualitatively represents the behavior of the correlation functions of the instantaneous speeds of the leukocytes (measured on the images time series), the deviation angle of their trajectory and their square displacement [10], [11], [23] is the Brownian Wormlike chain. In this model the single step azimuthal angle of the trajectory, θ (see **Fig 3A**), is distributed according to a Gaussian law with standard deviation 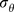, assumed to be 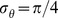 throughout the following studies of the leukocytes interactions. This is not the only model that can be adopted for the statistical description of the leukocyte motions in the absence of a stimulus. Alternatives to the WLC are, for example, the Ornstein-Uhlenbeck [40] and the Keller-Segel models [25]. All the studies presented in this report were performed by assuming a WLC-model for the un-stimulated leukocytes.

In the effort to simplify the extremely complex interplay of chemokines and their receptors, we assume that no chemokines are secreted by un-stimulated dendritic cells and that a single class of chemokines (CKs) is secreted under the action of an external microbial stimulus, such as CpG DNA or lipopolysaccharide. Representatives of this chemokines are CXCL9 and CXCL10 [17], two kinds of CXCR3 ligands. CXCR3 is a receptor expressed primarily on activated T limphocytes but also on the NK cells [17]. However, the interactions between the two classes of leukocytes, dendritic and NK cells, are actually much more complex than the picture depicted above. It is known, for example, that dendritic cells are made competent for the activation of NKs by a number of cytokines whose production is triggered by the external stimulus and also by feedback loops between NK and dendritic cells [5], [41]. The dynamics of these signal channels is simulated here by a smooth time variation of the secretion of chemokines by dendritic cells.

In summary, the whole process of interaction between NK and dendritic cells is studied here within a Brownian Dynamics approach. The NK cells diffuse around fixed DCs under the action of a mean field leukocyte-leukocyte interaction force, indicated as 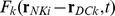 in **Eq.8**. These forces are derived from a model that describes the production, the diffusion and the degradation of the chemokines (**Fig 3B** and **Fig** **2**). One of the aims and the results of this report is the analytical formulation of this interaction force derived under suitable approximations and implement it in a mixed Brownian Dynamics/Monte Carlo algorithm (see **Fig** **2**).

To this purpose we work on the Keller-Segel model [25] with time dependent sources and show that, since the chemokine production by dendritic cells [1], [41], [42] and the chemokine diffusion occur on widely different time scales (few hours compared to minutes), the chemokine concentration can be written in a space-time separable form that can be used in simple Brownian Dynamics simulations.

In the Keller-Segel model one assumes that the cell dynamics is determined by an effective potential originated by the action of the chemokines, whose concentration is c (**r**,t), with efficacy measured by the chemotaxis sensitivity parameter, χ [25]. Within these assumptions the complete Fokker-Planck equation of a NK cell that diffuses under the action of the chemokines secreted by an immobile DC, acting as a chemokine source with current density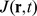, is [25], [27] (see **Fig** **1B**):
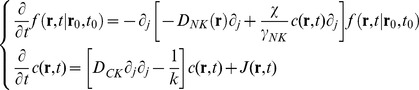
(9A)


The function 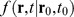 represents the probability to find the NK cell at the distance **r** from the dendritic cell at time t, when it was at the distance **r**_0_ at the time t_0_. The chemokines diffuse with the diffusion coefficient D_CK_. The aim of these coupled equations (Eq.9) is to describe the motion of the NK cells as pure random diffusion in the presence of a drift force that depends on the gradient of the chemo-attractants. Eq.9B closes the system by describing the evolution of the chemo-attractants as determined by random diffusion (with diffusion coefficient D_CK_) superimposed to a degradation process with characteristic time *k*. A variety of functional forms for χ and *k* have been proposed and adopted for applications in the case of bacteria motility [43]. We assume here for simplicity that the chemokines' degradation rate, *1/k,* is independent of the cell concentration (correct in the limit of low concentration of the dendritic cells or low inflammation conditions) and that the chemotactic parameter χ does not depend on the chemokine concentration c (**r**,t). This assumption is valid here and for a production of chemokine that is about one order of magnitude larger. In fact, by taking 10 dendritic cells per box, 500×500×50 μm^3^ in size, an average value N_CK_ ≅10^5^ of chemokines per dendritic cell (see **Table** **1**) and *k* values in the range 300–600 s the concentration of CKs is approximately n_CK_  = 1.4 nM. The encounter rate of the chemokines on the NK cells can be computed by means of the relation [44] 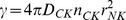 (D_CK_ is the diffusion coefficient of the chemokines, n_NK_ is their concentration, and r_NK_ is the NK cell radius, see **Table** **1**). With the values adopted here for D_CK_  = 10 μm^2^/s and r_NK_  = 5 μm we compute a maximum chemokines consumption rate of approximately 0.2 s^−1^. This rate is two orders of magnitude less than the average rate of production of the chemokines, as estimated from N_CK_/τ ≅10^5^/(300 s) ≅330 s^−1^. Therefore our assumption that the degradation rate is not dependent on the cell concentration can be considered valid here and for dendritic cell concentrations at least one order of magnitude larger.

The chemotactic sensitivity, χ, is a measure of the effect of the chemical gradient on the cell motion [25], [45], and it can be positive (chemo-attraction) or negative (chemo-repulsion). In the limit D_NK_ 0, i.e. for vanishing diffusion of the NK cells, the Keller-Segel model describes a chemotactic collapse leading to aggregates or Dirac peaks. Here we want to describe instead a dynamic equilibrium in which NK cells follow the chemical gradient but are also able to escape the dendritic cell attraction basin.

Under the assumption that the NK distribution function derivatives are much smaller than the corresponding spatial derivatives of c (**r**,t), the Langevin equation that corresponds to **Eq.9A** is (see also **Eqs. 1, 2**):
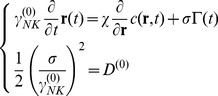
(10)


The term 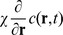 plays the role of an effective conservative force that corresponds to the potential function 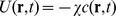 and equals therefore the force term 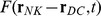 in the full dynamic equation (**Eq. 6**). The chemokine concentration is then the fundamental function to be computed since it determines the effective potential and the NK cell motion. Conversely, its evolution is determined by the chemokine source term, 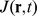. Therefore we first search for solutions of **Eq.9B** under different functional forms of the chemokine source term, J (**r**,t).

#### Interacting dendritic-NK cells

Two possible cases can be envisioned in which the emission of the chemokines by the source (the dendritic cell), characterized by the current density 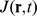, is decreasing (*interacting* case) or increasing (*noninteracting* case) in time. This can be due to feedbacks in the chemokines production by the dendritic cells interacting (*abbreviated to “inter.”*) or non interacting (*abbreviated to “non-inter.”*) with NK cells. In order to ensure the convergence of the solution, we have also to assume that the source is adiabatically switched on at t = 0 with a time constant τ. This parameter acts both as a mathematical tool and as the characteristic time response of the dendritic cell to the stimulus. For the *non interacting* case we write the current density as:
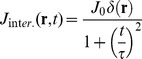
(11)


The solution of **Eq.9B** with this source term can be obtained by Fourier Transform methods (see paragraph S3,*“FT solution of the KS equation”* in the **SI**) and it is:
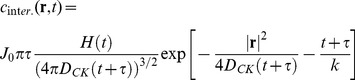
(12)


**Figure 4:**
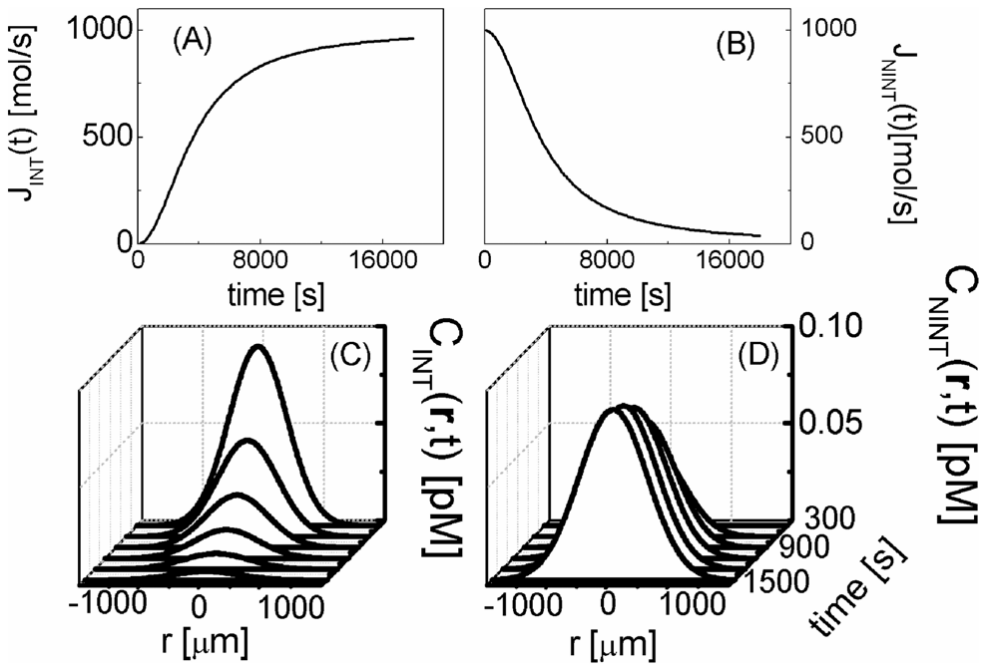
Evolution of the chemokine concentration. Current density 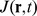 that describes the chemokines production by the dendritic cell when interacting (panel A, **Eq.11**) or non interacting (panel B, **Eq.13**) with a NK cell. Panels C and D report the time evolution of the spatial distribution of the concentration of the chemokines in the two cases of interacting (panel C) and non interacting (panel D) leukocytes. The chemokine source parameters were: J_0_ = 1000 mol/s; k  = 600 s; τ  = 3600 s; D_CK_ = 10 µm^2^/s (**Eq.12, 14**).

The function 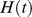 is the Heaviside function and the variance of this function increases as 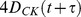. The concentration is decreasing with the functional form 
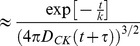
 from the maximum value 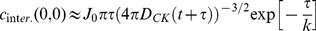 (see **Fig 4**).

When the dendritic and NK cells are not interacting, the source term decreases and it can be written as:
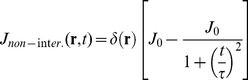
(13)


The solution of **Eq.9B** with this source term can be found by considering only the time varying part of **Eq. 13**, by applying the Fourier Transform to **Eq. 9B** and by restoring the positiviness of the solution by adding the concentration value at the source at t = 0. As a final result (see paragraph S3, “*FT solution of the KS equation*” in File S1 for details), we can write the chemokine concentration for the non-interacting case as:
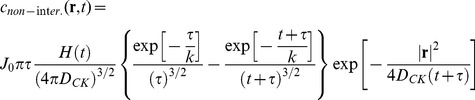
(14)


With this assumption, the maximum value of the chemokine concentration starts from 0 and follows an increasing exponential trend up to a plateau value, 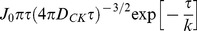, as shown in **Fig 4**.

The solutions given in **Eq.12** and **Eq.14** can now be used to derive the effective chemotactic interaction force, 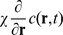, to be used as force (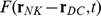) in **Eq. 6**. A critical point to this issue is that the chemokines concentration suffers two relaxation processes due to the diffusion of the chemokines themselves and to the variation in time of the source, *J* (**r**,t). This implies that in the solution of **Eq.9A** the diffusive motion of the chemokines is coupled to that of the NK cells.

### Constant source of chemokines

We first focus on the case of constant source of chemokines. This is an artificial situation in which there is no feedback between the two classes of leukocytes (here NK and dendritic cells). We want to develop a fast algorithm to simulate long trajectories of dendritic and NK cells. In order to avoid the integration of **Eqs.9** along with the Brownian dynamics steps of our algorithm we derived an approximated analytical expression for the chemokine concentration (see File **S1**, Eqs. S24 and S25 in paragraph S5: “*Numerical Approximations*”). With these approximations we can perform long numerical simulations of the interactions between NK and dendritic cells and study the effect of the chemotactic term 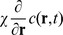 (see **Eq. 6**). Several simulations were run with different values of the dendritic cell sensitivity parameter χ and of the NK cells density (4000–8000 cells/mm^3^; 500x500x50µm^3^ simulation box volume) with integration time step Δt  = 1 s. The following additional parameters were also assumed (see also **Table 1**):
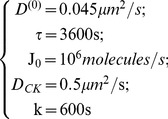
(15)


As discussed in **File S1** (paragraph S5, “*Numerical Approximations*”) this choice of parameters implies a ≅0.2% error on the chemokine concentration, due to the first order approximation derived in Eqs. S24 and S25 (in paragraph S5: “*Numerical Approximations*” of the SI).

The chemotactic parameter χ can vary widely depending on the specific system. The chemotactic attraction can indeed change by a factor as large as 10^2^ [18] varying from T cell (low χ) to dendritic and B cells (high χ), and corresponds to an average velocity 
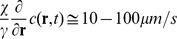
for in-vitro chemotaxis experiments such as the “under-agarose cell migration” experiments [26], [46]. However, the units of 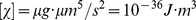 suggest us to obtain an estimate of χ from the average kinetic energy per volume according to the following scheme:
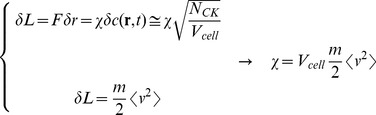
(16)where N_CK_ is the average number of chemokines produced per dendritic cell, m_NK_ is the NK cell mass and V_DC_ is the dendritic cell average volume (see also **Table 1**). The typical experimental value of the average value of the NK speed computed over time steps ≅1 s, is 0.2 µm/s [8]–[13], [37]. If we further assume an average value of chemokines per cell, N_CK_ ≈10^5^, we obtain for the chemotactic parameter the estimate 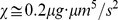. We have therefore decided to assume the range 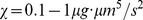 in the following explorative simulation study.

**Figure 5:**
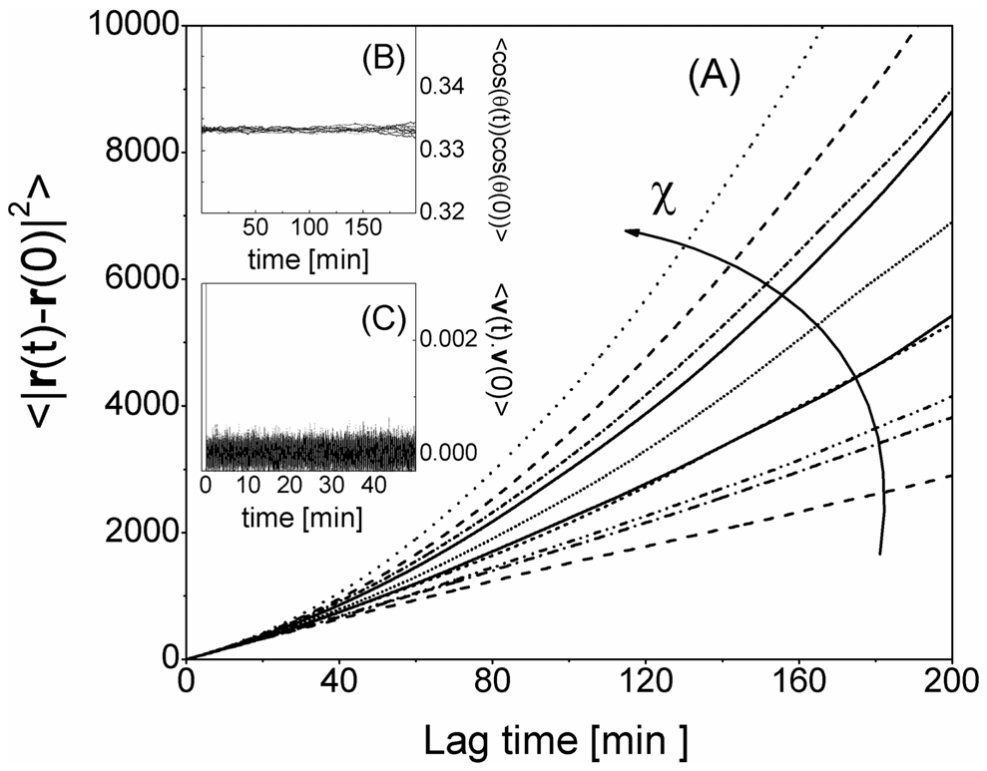
Autocorrelation functions for the NK cells under constant chemotactic force. Panel A: square displacement 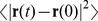. Panel B: the single step deviation angle 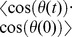. Panel C: the velocity autocorrelation function 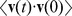. The results are averaged over 100 NK cells. The simulation parameters are reported in the text (**Eq.5** and **Table 1**). The increasing values of the chemotactic parameter are χ = 0.1, 0.2, ….. 0.9, 1.0 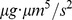.

For constant sources of chemokines (J (**r**)), the only relevant effect of the chemotactic term is on the RMS displacement (see **Fig 5**) that acquires the characteristic upward curvature also observed in the experiments [10]–[11], [23]. The curvature effect shown in **Fig 5** is overestimated since we are not allowing leukocyte-leukocyte interactions that would imply an alternating increase and decrease in the source strength, J (**r**) (see next paragraph), which is instead held fixed during the simulation. In the absence of leukocyte-leukocyte interactions, the chemotactic force contributes therefore with some additional directional motion of the NK cells, that is largely accounted for in our simulation procedure by the Brownian Worm like chain model (see S2,“*Un-stimulated lymphocyte stochastic motion within a lymph node*” in File S1).

### Metropolis algorithm for interactions

We now takes into account the feedback between the NK and dendritic cells. This is accounted for by modulating the production of chemokines by the dendritic cell based on the interaction with approaching NK cells. The chemotactic force,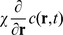, changes in time because of the diffusion of the chemokines away from a central dendritic cell and of the time behavior of the source (the dendritic cell). The production of the chemokines by the dendritic cell is inhibited by the contact with NK cells or stimulated by the absence of such interactions. This interplay of interaction and activation and de-activation of dendritic cells, is simulated by defining an interaction probability that is based on the distance between dendritic and NK cells and on the directionality of NK cell motion. This is a critical point of our study that we approach by devising a Monte Carlo test on a minimal condition represented by the direction of relative motion between a NK cell and the central dendritic cell.

The interaction (an attraction more than a direct contact) can occur only when the cells are within a threshold radius. This is a sensible assumption that is also motivated by the need to reduce the computation time. However, the probability of the interaction depends on the instantaneous concentration of the chemokines compared to its maximum value and it can occur also at a distance between dendritic and NK cells.

The basic idea on which we build the interaction algorithm is that a dendritic cell produces chemokines when it has not activated recently a NK cell. Otherwise the chemokine concentration around the dendritic cell decreases. The second basic idea is that the NK cell that approaches the dendritic cell feels the chemokine gradient formed around the dendritic cell (see **Eqs. 12** and **14**) or not, depending on the size of the displacement of the NK cell towards the central dendritic cell. The lower is the chemokine concentration around the dendritic cell, the larger must be the approaching leap of the NK cell towards the dendritic cell. The whole process is a feedback loop. When a NK cell starts an interaction, the chemokine concentration decreases around the dendritic cell. Therefore the probability that the dendritic – NK cell interaction comes to an end, increases. In this case the test on the interaction stop is made on the size of the NK leap away from the dendritic cell. The larger is the chemokine concentration on the dendritic cell, the larger must be the NK jump away from the dendritic cell in order that the interaction stops.

Taking into account these considerations we have built the following Metropolis algorithm.

On each time step the interaction test is run only for distances between NK and dendritic cells that are lower than a_0_ = 25 µm. If this condition is fulfilled, we compute 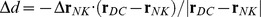, where 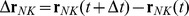 is the displacement of the NK cell on the simulation step, and consider two possible situations (see **Fig 3B, C**).

1. *Interaction_start.* If the interaction is not active, the chemotactic term is 
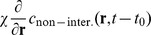
, where t_0_ is the time at which the previous interaction was stopped. The interaction begins at time t_1_, only if (we recall that 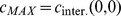)
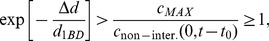
(17)where 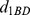
is the root mean square displacement with diffusion coefficient DNK: 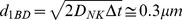. In this test the random variable is represented by the displacement Δd that contains the Gaussian distributed displacement of the NK cell. In such a case, from this time step on, the chemotactic term will be computed as 
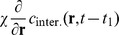
.
2. *Interaction_stop.* If the interaction is active, the chemotactic term is 
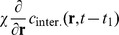
, where t_1_ is the time at which the interaction started. The interaction is interrupted only if

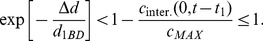
(18)It is noteworthy that if the NK cell is moving away from the DC, an interaction cannot be initiated because 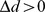, the Metropolis term is 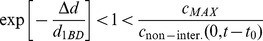 and the probability for the interaction to be stopped is determined by the chemokine concentration, via the term 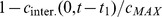. Symmetrically, if the NK cell is approaching a dendritic cell, an ongoing interaction has no chance to be interrupted since 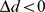 and the Metropolis term is 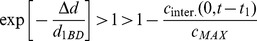. The probability for an interaction to be started in the case 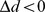 is proportional to the chemokine concentration, 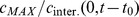.

**Eqs. 17** and **18** represent a Monte Carlo sampling in which the random number is embedded in the displacement Δd towards the dendritic cell. In this way we weigh the probability that an interaction starts on the direction of motion, i.e. whether NK cells are approaching a dendritic cell or not.

The overview of the proposed mixed Brownian Dynamics and Monte Carlo algorithm is shown in **Fig 2**. We test now the proposed solution of the KS model for the NK and dendritic cells in terms of number and duration of specific interactions.

### Simulation of the interactions between NK and dendritic cells

We consider two experimental situations that refer to the stimulated or unstimulated healthy murine system. Various types of stimuli can be applied to the organism to trigger the initiation of an immunoresponse to an antigen [47]. One of the most effective ones is the lipopolysaccharide, a component of the external wall of gram-positive bacteria [1]. This compound is recognized by the receptor complexes, including TLR4, CD14 and MD2, and elicits the production by DCs of several types of chemokines, including CXCL9 and CXCL10. We consider here for simplicity a single class of chemokines, though the whole simulation algorithm could be easily extended to a more complex situation. The dendritic cell activation time, τ in **Eqs. 12, 14**, could be modulated, in a more refined algorithm, by cytokines expressed also by NK cells such as TNFα [42], [48]. The diffusion parameter ensures that the average range of activity of the chemokines in the lymph node is 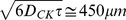.

**Figure 6:**
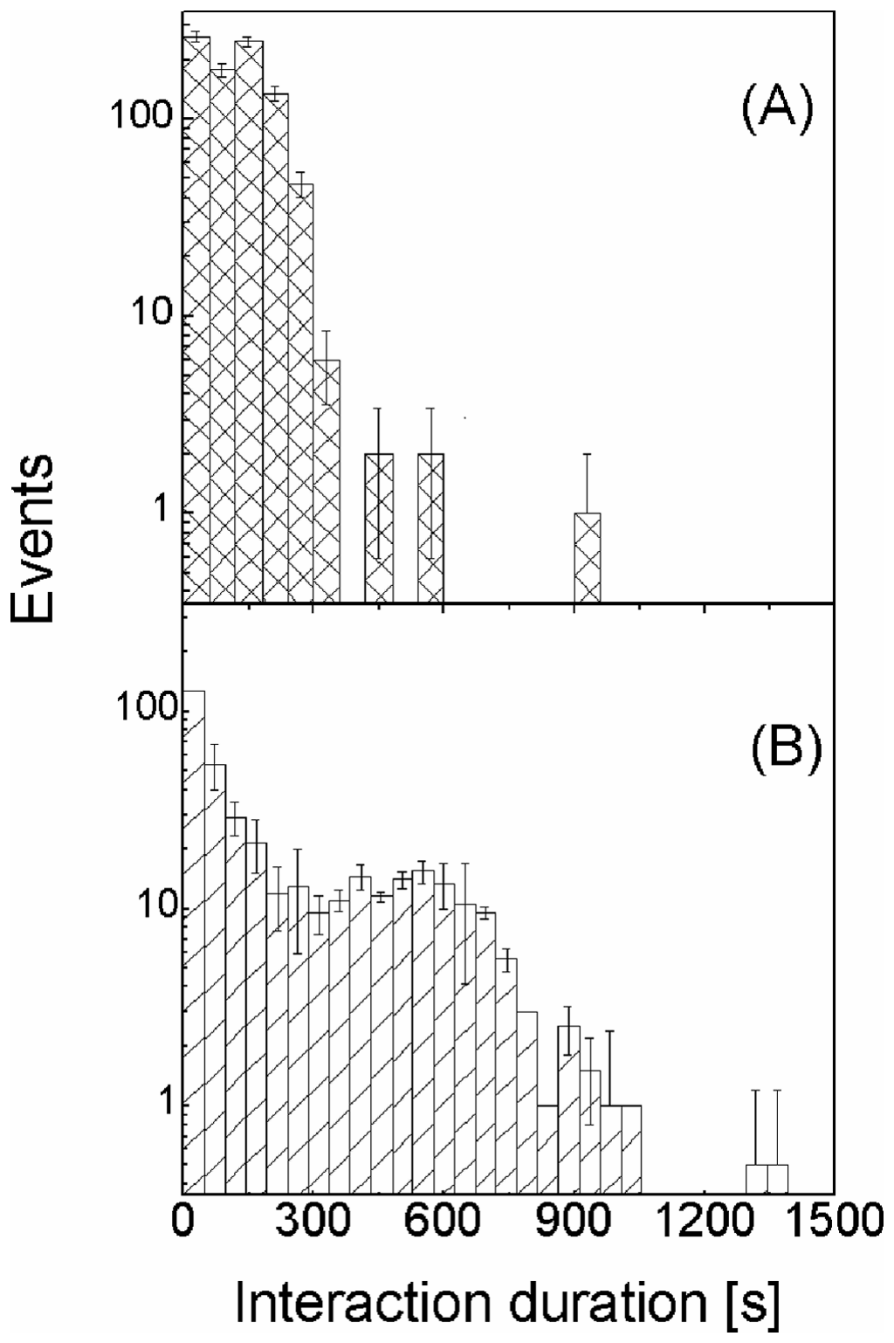
Distribution of the duration times of the interaction between NK and dendritic cells. Distribution of the duration times for the case of no chemotaxis (χ = 0, panel A) and of high chemotactic effect of the chemokines on the NK cells (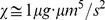, panel B). The results are obtained from the analysis of the interactions of 10 independent simulation runs (100 NK cells per simulation). The error bars are uncertainties obtained by 10 different simulations.

We have first run 10 simulations of non-stimulated dendritic cells (100 NK cells per simulation, χ = 0) and focused our analysis on the number and duration of the interactions as provided directly by the Monte Carlo tests (**Eqs. 17, 18)**. The histogram of the interactions' duration (**Fig 6A**) decreases rapidly with the duration: more than 90% of the interactions last for less than 300s. When stimulated dendritic cells are simulated under high chemotaxis conditions (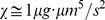), the distribution of the interactions' duration (**Fig 6B**) is much wider and only 60% of the durations last for less than 300 s.

**Figure 7:**
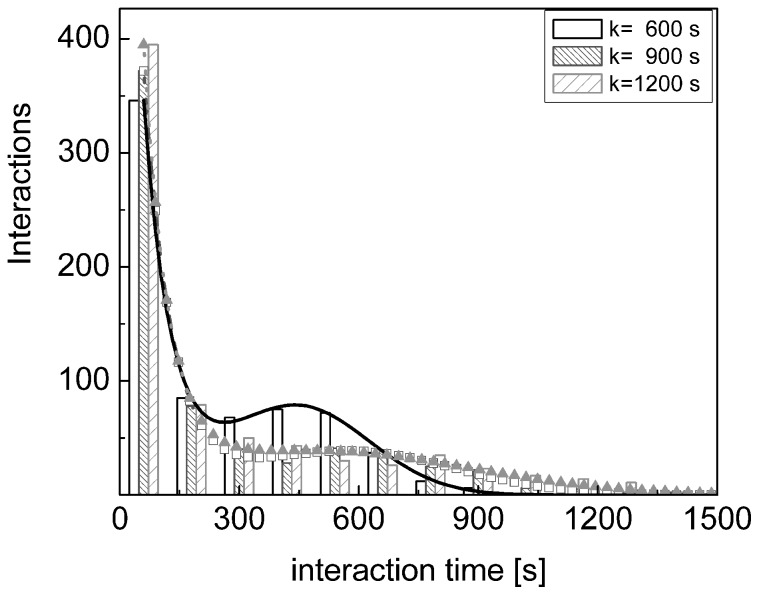
Dependence of the duration times of the interaction between NK and dendritic cells on the degradation time. Distribution of the duration time of the NK – dendritic cell interaction, as a function of the chemokines' degradation time, k: k = 600 s (open black bars, black continuous best fit line); k = 900 s (densely hatched red bars and red open squares best fit line) and k = 1200 s (sparsely hatched green bars and green filled triangles best fit line). The chemotactic parameter was set to 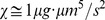. The lines are best fit to the trial function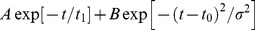. Best fit parameters are: A (600s)  = 803±28, t_1_ (600s)  = 70±6s, B (600s)  = 78±2, t_0_ (600s)  = 450±6 s, σ (600s) = 250±14 s for k = 600s; A (900s)  = 859±37, t_1_ (900s)  = 70±6s, B (900s)  = 38±2.5, t_0_ (900s)  = 570±30s, σ (900s)  = 380±40s for k = 900s and A (1200s)  = 980±70, t_1_ (1200s)  = 66±6s, B (1200s)  = 38±3, t_0_ (1200s)  = 630±200s, σ (1200s) = 550±90s.

A small systematic increase of the interaction probability is found at durations that are correlated to the value of the chemokines' degradation times *k*: the position of the secondary component at 440s–480s (**Fig 6**) changes slightly with the value of the degradation time, *k*, as can be judged from **Fig 7**. The position of the secondary peak in the distribution of the interaction durations moves to ≅580±40 s for *k* = 900 s (**Fig 7**, densely hatched bars and dashed red line). The width of this component also increases substantially with the degradation time and for *k* = 1200 s it is already not possible to visually single out a defined cell population with long interaction durations at finite values of duration times (**Fig 7**, sparsely hatched bars and dotted green line), though the presence of long interacting cells is evident from **Fig 7** for any value of k. On the other hand, in the case of very rapid (k<50–60 s) degradation of the chemokines we expect that the effect of non-contact interactions on the interaction duration would vanish.

We focus then our attention on the percentage of the NK – dendritc cell interactions with durations above a threshold value, T_t,,_ as done in most of the experimental studies [9], [12], [37], [49] and perform a simulation study as a function of the chemotactic parameter. This procedure allows us to model an increasing efficiency of the chemokines to attract NK cells and to study its effect on the number of long lasting interactions. In fact these interactions are likely related to the chemokine mediated recognition between leukocytes. From the distributions shown in **Fig 6** **,** **7** it is reasonable to set the threshold value for the long lasting interactions at T_th_  = 600s, the position of the secondary peak in the distribution of the duration times.

**Figure 8:**
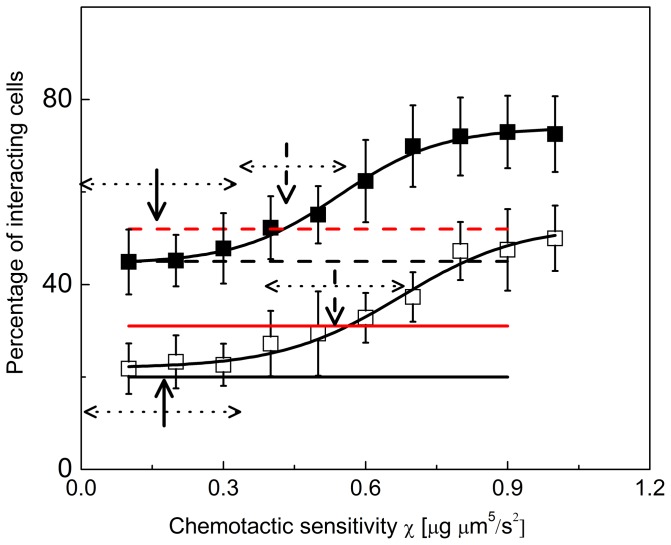
Dependence of the duration times of the NK – dendritic cell interaction on the chemotactic parameter. Percentage of the interacting cells computed on a set of 10 independent simulation runs. The percentage of all the interacting cells (filled squares) and of the long interacting cells (open squares; interaction duration larger than T_th_ = 600s) are reported. The lines are the sigmoidal fits to the data (y = 74–33.5/(1+ exp ((χ−0.54)/0.108)), solid line; y = 53–31/(1+ exp ((χ−0.674)/0.127)), dashed line). The horizontal lines indicate the experimental percentage of the NK cells interacting with dendritic cells: total number of interactions (dashed lines) under no stimulus (black) and under the action of lipopolysaccharide stimulus (red); long duration interactions (T_th_  = 600s) (solid lines) under no stimulus (black) and under the action of lipopolysaccharide stimulus (red). The vertical arrows indicate the most probable values of the chemotactic parameter as visually obtained from the intersection of the interpolating sigmoidal curves and the horizontal lines. The horizontal dotted arrows indicate the range of variability of the chemotactic parameter. The experimental data were obtained from experiments in ex-vivo lymph nodes from mice not treated with the lipopolysaccharide stimulus (black horizontal lines) and from mice treated with the lipopolysaccharide stimulus 4 hours in advance (red horizontal lines; see also *Materials and Methods*).

The total number of NK – dendritic cell interactions and the number of interactions with duration above T_th_, as a function of the chemotactic parameter χ, are summarized in **Fig 8**. The increase of the interactions' duration (total and long lasting interaction duration) with χ observed in **Fig 8** indicates that there is a definite relation between these two parameters and suggests that there is an effect of non-contact interactions between leukocytes on the measured interaction duration, typically taken as a fingerprint of ongoing contact interaction [8]–[13]. From a physical point of view it is clear that a central attraction towards the dendritic cell is required to sustain the touch and go behavior observed in most two-photon excitation in-vivo investigations of the leukocytes interaction [13], [37]. Considering the extremely high dilution of cells within a lymphoid organ, the absence of such a central attraction would determine a drift of a NK cell away from the central dendritic cell as soon as an interaction stops, therefore not allowing for a sustained patrolling of the dendritic cell surface in a quest for specific recognition.

The simulated values of the total number and of the long duration interactions agree qualitatively with experimental values. These were obtained from NK – dendritic cell interaction experiments in ex-vivo mice lymph nodes (**Fig 8**) under no stimulus (black horizontal lines) and when the mice are stimulated 4 hours in advance with lipopolysaccharide (red horizontal lines; see also *Materials and Methods*). By visually inspecting the intersection between the interpolating sigmoidal functions in **Fig 8** with the experimental values we can estimate two values of the chemotactic parameters, χ ≅ 0.15±0.15 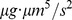 and χ ≅ 0.5±0.2 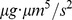 that describe simultaneously the long and total number of interactions under no stimulus and when lipopolysaccharide was injected in the mice.

## Discussion

The mean field model of non-contact interactions developed here indicates clearly some direct effect of these interactions on the interaction duration. But this main result might be invalidated by major flaws arising from the crudeness of our approximations. We have then carried out a validation of the numerical model employed here by comparing simulated and experimental data on an extended set of additional geometrical and kinematic parameters of the NK cells motion. To this purpose we have selected three parameters, namely the confinement ratio of the NK cell trajectory (C_R_, defined as the ratio of the distance run by a NK cell divided by the trace length), the instant 3D velocity (V_3D_) of the NK cells and the length of the cell trajectory (L_traj_). Details on the experimental conditions and the analysis of the experimental and simulated data are reported in File S1 (see paragraph S4 “*Analysis of dendritic and NK cells interactions*” in File S1).

**Figure 9:**
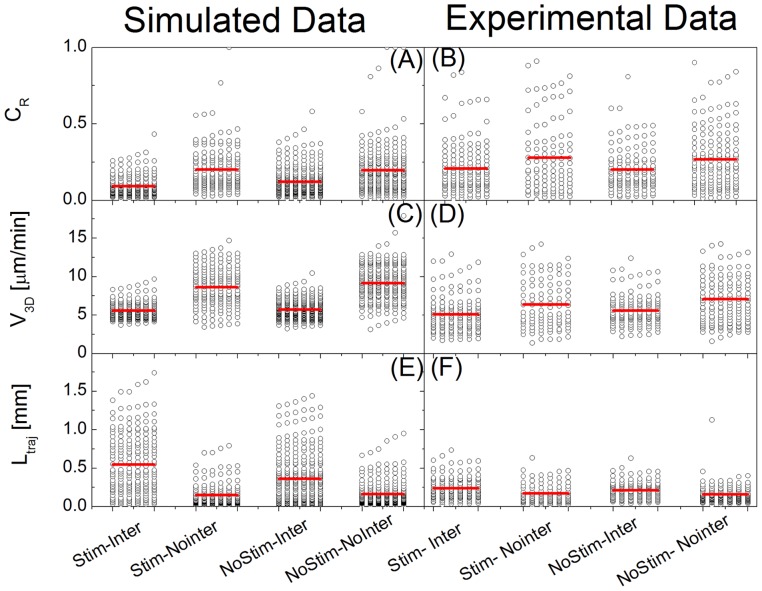
Result of the analysis of the simulated and experimental trajectories of NK cells. The simulated trajectories were obtained by assuming 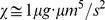 and were rebinned to reproduce the experimental time acquisition step (25′), as detailed in File S1 (paragraph S4:“*Analysis of dendritic and NK cell interactions*”). The NK cell confinement ratio (C_R_), the 3D speed (v_3D_) and the trajectory length (L_traj_) computed on more than 200 trajectories (NK cells) are reported in Panels A, B (confinement ratio), C, D (speed) and E, F (trajectory length). In each panel the labels “stimulated” and “un-stimulated” indicate the cases under stimulus (lipopolysaccharide injected i.v. in the mouse for the experimental data and 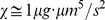 for the simulated data) and under no stimulus (no lipopolysaccharide injected i.v. in the mouse for the experimental data and 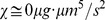 for the simulated data). Interacting and non-interacting NK cells were determined in the experimental and simulated data by the instantaneous NK cell velocity and the NK – dendritic cell distance, as described in the text. The horizontal bars indicate on each data set the average value.

Interactions on the experimental images were detected by requiring that (1) the distance between NK and dendritic cells is less than 25 μm, (2) the confinement ratio is decreasing during the putative interaction duration and (3) the instantaneous speed is below a threshold computed as the average of the instantaneous speed of all NK cells over all frames considered in a field of view (see paragraph S4,“Analysis of dendritic and NK cell interactions” in **File S1**). Interactions on the simulated data were detected as from the Monte Carlo tests (see **Eqs. 17, 18**). The result of this procedure, applied to ≅250 experimentally tracked NK cells and an equal number of simulated cells, is summarized in **Fig 9** for the stimulated (χ ≠0) and un-stimulated (χ = 0) case and for the subpopulations of interacting and non-interacting cells.

The confinement ratio accounts for the overall volume spanned by the NK cell during its motion and DC-NK cell interactions lead to its reduction (**Fig 9A, B**). The experimental data show wider distributions and a slightly higher average value than the simulated ones. However the relative interacting/non-interacting and stimulated/non-stimulated changes are similar in the simulated and experimental data. The lower simulated values may arise from a slight overestimation of the chemotactic parameter (see **Fig 8**) that in **Fig 9** is kept at 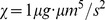.

Regarding the NK cell velocity (**Fig 9C, D**), the simulated interacting NK cells again show narrower distributions than the experimental data but the average values, <v_3D_>, of the NK cell velocity are very well reproduced. The average values <v_3D_> under no external stimulus are instead slightly larger than the experimental ones (**Fig 9C, D**), indicating that additional interactions of NK cells with the lymphoid tissue without any stimulus should be accounted for in a more refined model.

The trajectory length of the simulated NK cells provides an indirect estimate of the duration of the confined motion. The interacting NK cells spend much more time in patrolling the dendritic cell and in so doing drive longer paths. The presence of a very long tail in the trajectory length distribution is an indication of the overestimation of the chemotactic parameter assumed in **Fig 9** and/or an underestimation of the chemokine degradation rate k. However, the observation of longer trajectories for the stimulated NK cells, together with their lower average velocity, is an indication that our numerical mean field model grasps the main features of the intermittent interaction between NK cells and DCs, as found experimentally [13], [37]. The simulated non-interacting NK cells have instead a much shorter trajectory length, an indication of quasi-directional motion: simulated and experimental non-interacting NK cells display very similar distribution and average values of the trajectory length.

In summary, the aim of this study was to ascertain the possible effect of non-contact interactions between leukocytes on the leukocyte-leukocyte interaction duration under pathogenic stimuli. To this purpose we have focused ourselves on the recent report [50] of interactions between dendritic and NK cells. We have chosen to model the non-contact interactions between leukocytes by means of the chemotactic potential proposed by Keller and Segel [25]. Starting from their formulation, we have devised a space-time separable analytical approximated functional form for the chemotactic attraction potential and used it in numerical simulations of the interaction between leukocytes under the action of chemokines. In order to obtain the overall behavior of NK cells and DCs under the action of chemotactic (or a generic non-contact interaction) and contact interactions, we have further assumed to describe contact leukocyte interactions as a reduction of the diffusion coefficient of NK cells that approach DCs.

The analysis of the simulations and their comparison to experimental data, indicates that the mean-field description adopted to describe the non-contact and contact interactions allows us to grasp the main features of the observed NK cell motion within the lymph node and to bring into evidence the possible effect of non-contact interactions on the geometrical and kinematic parameters.

In particular, our simulations indicate that the number of interactions is well described by a smooth sigmoidal trend as a function of χ. The overall fractional increase in the number of interactions is only 1.4 for the long interactions and it saturates as the chemotactic parameter increases above χ ≅0.7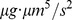. Such an increase is probably difficult to be detected on in-vivo or ex-vivo data also due to the experimental uncertainties and the intrinsic variability of the tissue surgical preparation. It is nevertheless a clear indication of a direct effect of non-contact interaction on the parameters typically measured to bring into evidence leukocyte-leukocyte contact interactions.

The analysis reported here suggests also that the discrimination between interacting and non-interacting leukocytes might be revised by taking into account also geometrical and kinematics parameters that are not related only to the leukocyte-leukocyte distance and therefore to their contact.

Finally, it is noteworthy that the reciprocal activating crosstalk between dendritic and natural killer cells has received recent attention in the literature [51]. Much interest has been focused on functional dendritic – NK cell crosstalk and its role in immune regulation [41], [52], [53]. For example, mature dendritic cells can activate NK cell cytotoxicity and IFN-γ production [42]. Conversely NK cells can enhance dendritic cell maturation and immune-stimulatory capacity. In particular several studies have reported that NK cells markedly increase their capacity to produce proinflammatory cytokines [3]. Therefore dendritic and NK cells seem to guide each other's functions not only through cell–cell contact but also through the release of soluble factors, including cytokines [51]. To this regard the possibility to study in vivo or ex-vivo in lymph nodes the role of the chemokine mediated interactions is becoming increasingly important.

## Supporting Information

File S1 All supporting information has been included in the Supporting Information file that covers the following contents: S1, Test of the Random Number generator. S2, Un-stimulated lymphocyte stochastic motion within a LN. S3, FT solution of the KS equation. S4, Analysis of DC and NK cell interactions. S5, Numerical Approximations.
(DOCX)
