# Supplementary material for: Modeling Leukocyte-Leukocyte Non-Contact Interactions in a Lymph Node
Source: PLoS One. 2013 Oct 28;8(10):e76756. doi: 10.1371/journal.pone.0076756 (PMC3810259; doi:10.1371/journal.pone.0076756)
Supplement: File S1 — All supporting information has been included in the Supporting Information file that covers the following contents: S1, Test of the Random Number generator. S2, Un-stimulated lymphocyte stochastic motion within a LN. S3, FT solution of the KS equation. S4, Analysis of DC and NK cell interactions. S5, Numerical Approximations. (DOCX) [file pone.0076756.s001.docx]

**Supporting Information.**

Keller-Segel modeling of leukocyte-leukocyte

non-contact interactions in a lymph node.

Nicola Gritti^1^, Michele Caccia^1^, Laura Sironi^1,3^, Maddalena Collini^1^, Laura D’Alfonso^1^, Francesca Granucci^2^, Ivan Zanoni^2^, Giuseppe Chirico^1,*^.

**S1. Test of the Random Number generator.**

**S2. Un-stimulated lymphocyte stochastic motion within a LN.**

**S3. FT solution of the KS equation**.

**S4. Analysis of DC and NK cell interactions**

**S5. Numerical Approximations**

**S1: Test of the Random Number generator.**

The Brownian Dynamics (BD) algorithm solves the Langevin equation in the absence of inertial terms by computing the finite difference equation, **Eq. 1**. This is implemented by generating a Gaussian displacement$\Re(t,\Delta t)$, sampled from a Gaussian distribution $P\left(\Re(t,\Delta t)\right)$, which is based on pseudo-random generators [Press 2007]. In order to run long BD simulations (total number of steps 10^6^ - 10^7^) it is essential to minimize the correlations in the Gaussian distribution that is sampled numerically. We have verified this by integrating **Eq.1** for a cell diffusion coefficient D=0.045 μm^2^/s and for a maximum number of steps (Δt=1s) M = 10^7^. The results are shown in **Fig.S1**. The slope, determined from a simulation of the motion of 100 NK cells freely diffusing in the volume, corresponds to a diffusion coefficient $D=\left\langle\left|\mathbf{r}\left(t+\tau\right)-\mathbf{r}\left(t\right)\right|^{2}\right\rangle_{t}/(6\tau)=0.046\pm0.002$μm^2^/s, in excellent agreement (2±4%) with the input value. The exemplary data reported in **Fig. S1** correspond to a discrepancy of less than 4% at lag times ≅ 40 min.

**Figure S1**. Results of the simulation of 100 NK cells freely diffusing in a volume (500x500x50 μm^3^): root mean square displacement $\Delta^{2}=\frac{1}{N}\sum_{i=1}^{N} \left\langle\left|\mathbf{r}_{i}(t+\Delta t)-\mathbf{r}_{i}(t)\right|^{2}\right\rangle_{t}$versus lag time. The number of steps is 10^7^, Δt = 1s and D_NK_ = 0.045μm^2^/s. The solid line represents a best linear fit to the data (slope/6 = D = 0.046 ± 0.002 μm^2^/s). The inset reports the correlation function on larger time scales to bring into evidence the increase in the uncertainty with the simulation time.

**S2. Un-stimulated lymphocytes stochastic motion within a LN.**

A lymphocyte that diffuses in the lymph node experiences a number of interactions with different components of the tissue. The overall motion cannot be completely described by the Brownian Dynamics theory. Even in the absence of interactions between lymphocytes, it is likely that a lymphocyte undergoes a high number of temporary interactions with vessels and actin filaments, that may lead to a motion that is only at a first qualitative analysis Brownian, but in fact has a definite directional component. A quantitative assay of the lymphocytes’ motion passes through the analysis of the root mean square displacement of the cells and the end-to-end vector analysis of their tracks (faster migration, orthotaxis; preferential turning, topotaxis; increased persistence, klinotaxis).

This type of analysis accounts for the observed low mobility of the DCs and the partially oriented motion of the T and NK cells, topics that have been risen experimentally [Bousso 2008] and partially addressed theoretically [Textor 2011]. The motion of the un-stimulated lymphocytes can be partially characterized by three correlation functions: the correlation function of the instantaneous velocities, of the deviation angle of the trajectory between adjacent steps and of the square displacement. These are defined as:

$\begin{matrix} C_{VV}(\tau)=\left\langle\mathbf{v}\left(t+\tau\right)\cdot\mathbf{v}\left(t\right)\right\rangle_{t} \\ C_{\cos-\cos}(\tau)=\left\langle\cos\left(\theta\left(t+\tau\right)\right)\cos\left(\theta\left(t\right)\right)\right\rangle_{t} \\ RMSD(\tau)=\left\langle\left|\mathbf{r}\left(t+\tau\right)-\mathbf{r}\left(t\right)\right|^{2}\right\rangle_{t} \end{matrix}$ (S1)

Experimentally one samples the cell motion over a finite time step, Δt, and the cell velocity is computed as the finite displacement over this time step: it therefore depends on the image sampling time and the tracking time step.


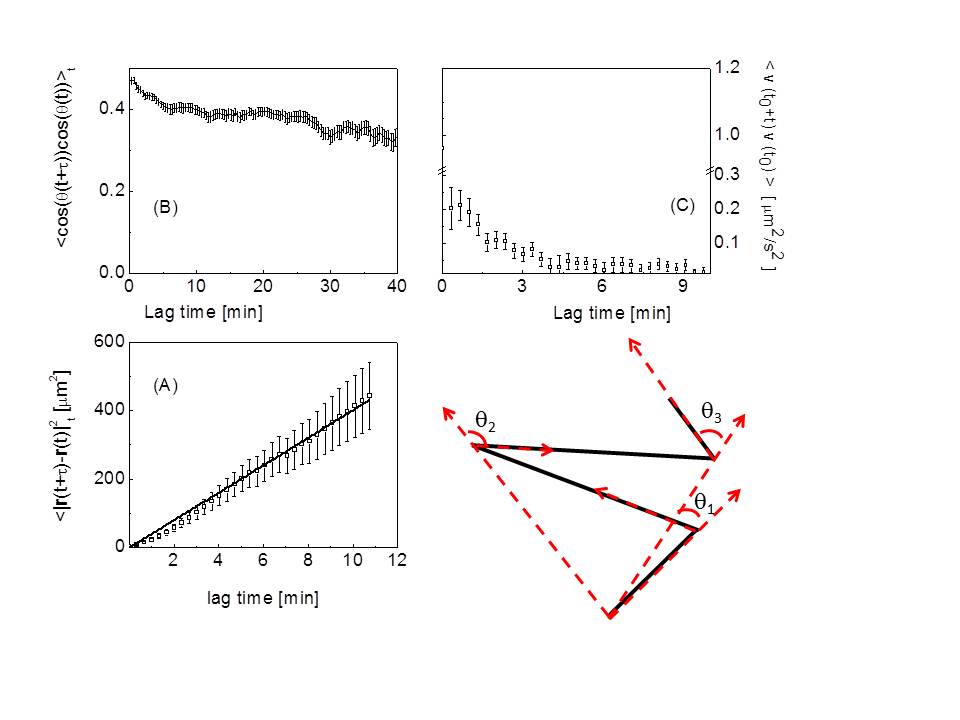


**Figure. S2.** Typical behavior of the experimental correlation functions, $RMSD(\tau)$ (panel A), $C_{\cos-\cos}(\tau)$ (panel B) and $C_{VV}(\tau)$(panel C, see **Eq.S1**), measured on NK lymphocytes diffusing in murine lymph nodes under no external stimulus (average computed over 70 NK cells). The solid line reported in panel A corresponds to the best linear fit to the experimental data. Panel B reports the deviation angle correlation function that for a pure Browinian diffusion should be constant at 1/3. The discrepancy between the data and the fit is particularly evident at small lag times (≤ 3-4 min). In panel C the velocity correlation function is reported with a break in the y-axis in order to get rid of the shot noise term. The sketch defines graphically the deviation angle θ(t).

The typical experimental behavior for lymphocytes diffusing in lymph nodes under no external stimulus is summarized in **Fig.S2** (for experimental details on the image acquisition see “*Two photon microscopy*” in M&M). The root mean square displacement shows a linear behavior for large lag times, as expected for a pure Brownian motion, but rounds off at small lag times as in a pure drift motion (**Fig. S2-A**). The autocorrelation function of the deviation angle, θ, shows (**Fig. S2-B**) particularly slow relaxing correlations. The instantaneous velocity shows a correlation on a finite number of lag times (**Fig. S2-C**), contrary to the pure Brownian motion for which a complete loss of memory of the velocity correlation function is expected. Different models can be employed to simulate these dynamic behaviors. We cite for example the Worm-Like Chain (WLC) model, in which the lymphocyte Brownian motion is constrained on a worm-like chain, in a way similar to what done in [Klenin 1998], and the Ornstein-Uhlenbeck model, that takes into account velocity correlations [Uhlenbeck 1930]. It is worth noting here that also the Keller-Segel model [Keller 1971] could provide qualitative agreement of the observed dynamics as discussed in the main text (see **Fig.3**).

In order to perform a Brownian Dynamics simulation on a worm-like chain we begin from the pure Brownian Dynamics assumptions and implement the Brownian Dynamics steps on an environment that constrains the cells to move in a preferential direction. This preferential direction of motion is not due to a chemotactic effect, since experimental RMSD plots with a non-Brownian behavior (i.e. non quadratic trend for short lag times) appear both for interacting and non interacting cells. In order to describe such a preferential direction of motion, we have to sample the cell displacement in polar coordinates. We can imagine to sit on a cell and align its motion along one axis that defines the persistence direction. This algorithm tries to simulate the anisotropy of the lymph node tissue that is described in patches characterized by different persistence axes (see the sketch in **Fig. S2**). We modify the azimuthal distribution function from $f(\theta,\tau;0,0)=\pi^{-1}$to:

$f(\theta,\tau;0,0)=\frac{1}{\sqrt{2\pi\sigma_{\theta}^{2}}}\exp\left[-\frac{\theta^{2}}{2\sigma_{\theta}^{2}}\right]$ (S2)

The most likely azimuthal angle is θ = 0, that corresponds to the direction of motion, and the variance $\sigma_{\theta}^{2}$ is a measure of such directionality. The limit $\sigma_{\theta}^{2}\to\infty$ is equivalent to a pure random walk, whereas in the limit $\sigma_{\theta}^{2}\to0$, the WLC model approaches to a pure drift motion. The results of 4 simulations on 100 cells located in a box of 500x500x50 m^3^ with boundary conditions, are shown in **Fig. S3**. The Worm Like Chain model describes qualitatively the trends of all the three correlation functions defined in **Eq. S1**. However, it was not possible to find a single value of the variance that enables to quantitatively describe simultaneously all the correlation functions. The trend observed in the experimental correlation functions depends also on the acquisition time step and on the experimental conditions, also related to the state of the lymph node. Moreover we are interested here in simulating the chemotactic effect. Therefore we limit ourselves to a qualitative description of the un-stimulated case, that we obtain within the worm-like model with $\sigma_{\theta}=\pi/4$.

**Figure S3.** Correlation functions (see **Eq. S1**) computed on the four different numerical simulations (100 cells per simulation) according to the WLC algorithm. Panel A: root mean square displacement, RMSD(t). Panel B: correlation function of the cosine of the deviation angle. Panel C: correlation function of the instantaneous velocity. The variance of the azimuthal probability function increases along the arrows: $\sigma_{\theta}=\pi/80;\pi/8;\pi/4;\pi/2$.

**S3. FT solution of the KS equation**

In order to solve the Keller-Segel model, **Eq.6B**, we need to specify the source term J(x;t). We first assume the source to be caused by one fixed DC in the origin of the axis. We have two possible cases. If the DC is interacting with one or more NK cells, the source is decreasing in time because the reservoir of chemical agent is emptying out. If the DC is non interacting, the source term is increasing in time, allowing the nearby NK cell to feel the presence of the non interacting DC. For convergence reasons, we have also to assume the so called adiabatic approximation, i.e. the source term must be slowly increasing for t < 0.

Let us assume a first simple space-time dependence of the source that is, a point source whose amplitude changes with time as a Lorentzian function:

$J_{INT}(\mathbf{r},t)=\frac{J_{0}\delta\left(\mathbf{r}\right)}{1+\left(\frac{t}{\tau}\right)^{2}}$ (S3)

Here J_0_ represents the emission rate of chemical agents by the source. Notice that for t < 0, the Lorentzian function is slowly increasing, while, for t > 0, the source is decreasing with half-life ≅ *k*. **Eq.6B** then reads:

$\partial_{0}c(\mathbf{r},t)=\left[D_{CK}\partial_{j}\partial_{j}-\frac{1}{k}\right]c(\mathbf{r},t)+J_{0}\frac{\delta\left(\mathbf{r}\right)}{1+\left(\frac{t}{\tau}\right)^{2}}$ (S4)

One can solve this differential equation with Fourier Transform. The source term becomes:

$\tilde{J}_{INT}(\mathbf{q},\omega)=\int\int d\mathbf{r}dt\exp\left[-i\mathbf{q}\cdot\mathbf{r}+i\omega t\right]J_{INT}(\mathbf{r},t)=J_{0}\pi\tau\exp\left[-\left|\omega\right|\tau\right]$ (S5)

The differential **Eq.C.2** becomes then in the Fourier space as follows:

(S6)

The solution is then:

$\tilde{c}(\mathbf{q},\omega)=\frac{J_{0}\pi\tau\exp\left[-\left|\omega\right|\tau\right]}{i\omega+\left[q^{2}D_{CK}+\frac{1}{k}\right]}$ (S7)

The latter identity is then anti-trasformed by means of the Residue Theorem around the pole

$\omega_{0}=i\left[q^{2}D_{CK}+\frac{1}{k}\right]$ (S8)

as in **Fig. S4**, thereby obtaining the solution in the direct space:

$c_{INT}(\mathbf{r},t)=J_{0}\pi\tau\frac{\theta\left(t\right)}{\left(4\pi D_{CK}\left(t+\tau\right)\right)^{3/2}}\exp\left[-\frac{\left|\mathbf{r}\right|^{2}}{4D_{CK}\left(t+\tau\right)}-\frac{t+\tau}{k}\right]$ (S9)

**Figure. S4.** Sketch of the integration path for the anti-transformation of **Eq. S7**.

We notice that as τ approaches zero, the initial condition on the source becomes a delta function:

$\lim_{\tau\to0} J(\mathbf{r},t)=J_{0}\delta(t)\delta^{3}(\mathbf{r})$ (S10)

The solution in this case is:

$c_{INT}(\mathbf{r},t)=J_{0}\frac{1}{\left(4\pi D_{CK}t\right)^{3/2}}\exp\left[-\frac{\left|\mathbf{r}\right|^{2}}{4D_{CK}t}-\frac{t}{k}\right]$ (S11)

which is the solution of the diffusion-degradation operator:

$L_{diff-\text{degrad}}=\frac{\partial}{\partial t}-D_{CK}\partial_{j}\partial_{j}+\frac{1}{k}$ (S12)

For the case of non interacting lymphocytes the source is increasing with time as the opposite of a Lorentzian function, according to the following functional form:

$J_{NINT}(\mathbf{r},t)=J_{0}-\frac{J_{0}\delta\left(\mathbf{r}\right)}{1+\left(\frac{t}{\tau}\right)^{2}}$ (S13)

The Fourier transform of this function is:

$\tilde{J}_{NINT}(\mathbf{q},\omega)=\int\int d\mathbf{r}dt\exp\left[-i\mathbf{q}\cdot\mathbf{r}+i\omega t\right]J_{NINT}(\mathbf{r},t)=-J_{0}\pi\tau\exp\left[-\left|\omega\right|\tau\right]$ (S14)

The solution in the Fourier space is:

$\tilde{c}_{NINT}(\mathbf{q},\omega)=-\frac{J_{0}\pi\tau\exp\left[-\left|\omega\right|\tau\right]}{i\omega+\left[q^{2}D_{CK}+\frac{1}{k}\right]}$ (S15)

The solution in the real space is then the opposite of that obtained for the interacting case. The constant negative sign of this solution is not physically sensible and it is due to the fact that the source is not a module integrable function,$L_{1}\left(R\right)$. The solution to this problem is to sum to **Eq. S15**  a constant value, namely the value of $c_{NINT}(\mathbf{r},0)$, therefore obtaining a positive defined concentration for the chemokines also for the non-interacting case:

$c_{NINT}(\mathbf{r},t)=J_{0}\pi\tau\frac{\theta\left(t\right)}{\left(4\pi D_{CK}\right)^{3/2}}\left\{\frac{\exp\left[-\frac{\tau}{k}\right]}{\left(\tau\right)^{3/2}}-\frac{\exp\left[-\frac{t+\tau}{k}\right]}{\left(t+\tau\right)^{3/2}}\right\}\exp\left[-\frac{\left|\mathbf{r}\right|^{2}}{4D_{CK}\left(t+\tau\right)}\right]$ (S16)

**S4. Analysis of dendritic and NK cell interactions**

*Movie acquisition and tracking algorithm.*

The acquisitions contained a variable number (up to 500) of 3D volumes in which each stack was a 2D 256×256 pixels image. The z (optical axis) direction was sampled with 7-10 frames at a distance of 5 μm. The cell positions were interpolated in the z direction by weighting the intensity on each adjacent plane. The x-y field of view varied between 460×460 μm^2^ and 700×700 μm^2^.

The volume acquisition time (the minimum time between two xy frames at the same z height) was 10 - 30 s, depending on whether a Kalman filter (up to 5 frames) was applied during the acquisition. The cell motion was followed for 30-90 min under the two-photon excitation microscope. Cells were segmented and tracked by means of the program Volocity (Perkin-Elmer). Both NK cells and DCs were tracked in a 3D volume representing a large fraction (up to 700×700×50 μm^3^) of the lymph node. The frames in which a selected cell could not be segmented were disregarded during the analysis.

To perform cell tracking, we selected ROIs in the lymph node in which the density of cells (NK cells and DCs) allowed the discrimination of individual cells over time. The selection of the pool of NK cells that may interact with a selected DC was made according to a proximity consideration. Though DCs displayed much less motility than NK cells, we found necessary to keep track of their position over time in order to evaluate correctly the dynamic parameters (see below) that were used to estimate the duration of the interactions.

For simulated trajectories, we re-binned the time step (simulation time step = 1 s) to the experimental time step (average time step = 25 s) with a spline routine as exemplified in **Fig. S5**.


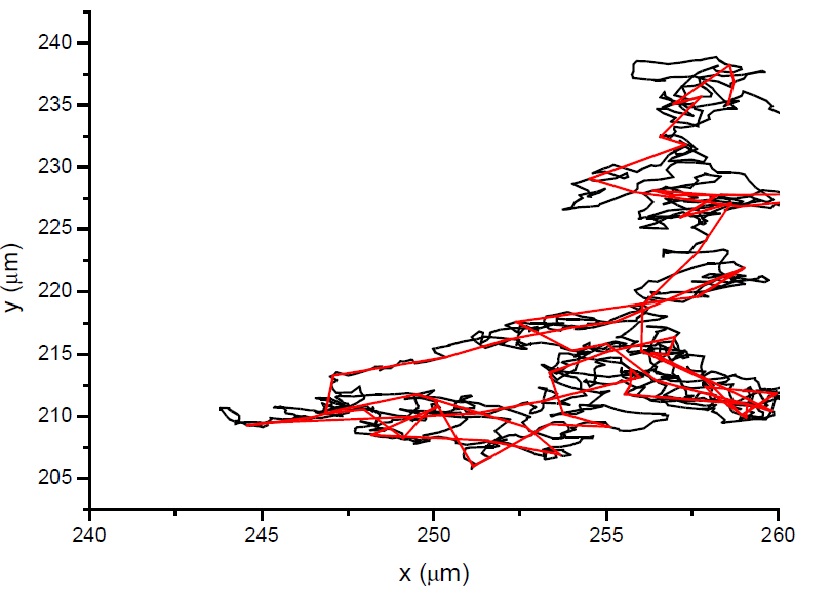


**Figure S5.** Example of a simulated trajectory (simulation time step 1 s) re-binned to the experimental time step 25 s by a B-spline curve.

*Computation of kinematic parameters.*

To characterize cell trajectories, we have computed and combined three parameters: the instantaneous velocity, the confinement ratio and the NK-DC distance. The rationale behind the choice of these parameters is that cell-cell interaction is mediated by the emission of chemical signals, implying that NK cells lie in close proximity to a partner DC for a finite time. Hereafter, we briefly describe the definition and the meaning of these parameters.

*NK-DC distance.* The mutual distance, *Dist(t),* between a NK cell and a DC can be used to assess if an interaction is occurring between the two cells. The threshold of NK-DC distance discriminating interacting and non-interacting events can be easily determined by looking at the size of NK cells (diameter 5-10 µm) and DCs (15-20 µm). For the latter, we should also consider the additional extension of dendrites (>10 µm [Bancherau 1998]). Taken together, we come to an estimate of D_cutoff_ = 25 µm as cut-off value for the NK-DC distance. If the distance between a NK cell and a DC lies below D_cutoff_ for the frames immediately preceding and following the i-th frame, then the two cells may be interacting in the i-th frame:

(S17)

For visualization purposes only, NK-DC distances can be digitized as follows:

(S18)

*Instantaneous velocity, v(t).* This parameter gives an estimate of the stepwise change in position of the cell (mainly NK cells) between consecutive frames. We expect that interacting cells display a reduced value of v(t) when compared to non-interacting cells. However, it must be considered that NK cells may constantly scan the DC surface, searching for receptors, even during productive interactions. As such, NK cells may still move around DCs at a significant speed, despite exploring a small space around the partner DC. As a result, the instantaneous speed may not be markedly decreased during the interaction. Consequently, while very fast or very slow events could be interpreted as non-interacting and interacting, respectively, with reasonable confidence, a "gray area" of medium-slow events would comprise both true and false positive interacting events. The instantaneous speed, taken alone, would not be able to discriminate between the two, but it would still be helpful in filtering out a number of truly non-interacting events.

A conservative value of the threshold, , can be selected by averaging the instantaneous speed of all NK cells over all frames considered in a field of view (or movie). We label the i-th frame as a putative NK-DC interaction if the instantaneous speed lies below the threshold value in the immediately preceding and following frames:

(S19)

*Confinement ratio.* Interacting NK cells, in addition to slightly reducing their instantaneous speeds, lie in a confined volume around the DC for a finite time. To model this, we borrowed the concept of end-to-end distance of a polymer from materials science, and applied it to cell traces. The cell position is defined by a 3D time vector = , where t_i_ is the time corresponding to the i-th frame and M is the number of frames over which the NK cell was tracked. We define the instantaneous displacement (analogous to the end-to-end distance of a polymer of i monomers) as:

(S20)

The instantaneous contour length of the track is defined as:

(S21)

The confinement ratio, d(t), is then defined as the ratio of the displacement over the contour length:

(S22)

In the main text we have reported the average value of the confinement ratio over the whole trajectory of the specific NK cell to characterize its trajectory:

$C_{R}=\left\langle d(t)\right\rangle_{t}$ (S23)

**S5. Numerical Approximations.**

In order to keep the complexity of the code and the CPU time at reasonable levels, we want to separate the spatial and the time dependence of the chemokine concentration to be used in the computation of the chemotactic potential. We use Brownian Dynamics methods to simulate the spatial diffusion of the NK cells and a Monte Carlo algorithm to account for the time fluctuations of the chemokine source. To this purpose, we assume that the diffusion characteristic time of the chemokines is negligible with respect to the characteristic response time, τ, of the dendritic cells. A visual inspection of **Fig.3** corroborates this assumption.

We want to develop a fast algorithm to simulate long trajectories of dendritic and NK cells. In order to avoid the integration of **Eqs.9** (see main text) along with the Brownian dynamics steps of our algorithm we decide here to write an approximated analytical expression for the chemokine concentration. We expand then the chemokine concentration in time starting from the maximum value of the chemical concentration with the assumption that the integration time step, Δt, is much smaller than the source relaxation time τ:

$\begin{matrix} c_{\text{non}-\text{inter.,inter.}}(\mathbf{r}_{NK}-\mathbf{r}_{DC},t_{0}+\Delta t)= \\ \text{ }=\left\{c_{\text{non}-\text{inter.,inter.}}(0,t_{0})+\left[\frac{\partial}{\partial t}c_{N\text{non}-\text{inter.,inter.}}(0,t_{0})\right]\Delta t\right\}\exp\left[-\frac{\left|\mathbf{r}_{NK}-\mathbf{r}_{DC}\right|^{2}}{4D_{CK}\tau}\right] \end{matrix}$ (S24)

The derivative of the chemokine concentration can be computed from **Eqs.12, 14** (see main text) and written in terms of $c_{\text{inter.}}(0,t)$, $c_{\text{non}-\text{inter.}}(0,t)$ and $c_{MAX}=Max\left\{c_{\text{inter.}}(0,t)\right\}_{t}=c_{i\text{nter.}}(0,0)$:

$\left\{\begin{matrix} \frac{\partial}{\partial t}c_{\text{inter.}}(0,t) & = & -c_{i\text{nter.}}(0,t)\left[\frac{3}{2\left(t+\tau\right)}+\frac{1}{k}\right] \\ & & \\ \frac{\partial}{\partial t}c_{\text{non}-\text{inter.}}(0,t) & = & \left(c_{MAX}-c_{\text{non}-\text{inter.}}(0,t)\right)\left[\frac{3}{2\left(t+\tau\right)}+\frac{1}{k}\right] \end{matrix}\right.$ (S25)

It is noteworthy that Eqs.**12** and **14** contain an Heaviside function to represent the switching between the states that correspond to interacting and non-interacting cells. This discontinuity implies the presence of a delta function of time, ~δ(t)(t+τ)^-3/2^, in the time derivative of Eqs. **12** and **14** that is taken into account by the choice of the concentration coefficients in Eq. **S25**, $c_{\text{inter.}}(0,t)$and $c_{MAX}-c_{\text{non}-\text{inter.}}(0,t)$.

Simulations were run with different values of the dendritic cell sensitivity parameter χ and of the NK cells density (4000 -- 8000 cells/mm^3^; 500x500x50 μm^3^ simulation box volume) with integration time step Δt = 1 s. The following additional parameters were also assumed (see also **Table I**):

$\left\{\begin{matrix} \text{ }D^{(0)}=0.045\mu m^{2}/s\text{ ; } \\ \text{ }\tau=\text{3600 s; } \\ \text{ J}_{0}=10^{6}molecules/s;\text{ } \\ \text{ }D_{CK}=0.5\mu m^{2}\text{/s;} \\ \text{ k}=\text{600s } \end{matrix}\right.$ (S26)

This choice implies that the maximum relative contribution of the first order term (**Eq. S25**) to the chemokine concentration is only $\cong\left[\frac{3}{2\tau}+\frac{1}{k}\right]\Delta t\cong0.002$, therefore supporting our first order approximation assumption.

**References of the Supplementary Materials.**

1. Bancherau, J.; Steinman, RM. Dendritic cells and the control of immunity. Nature 392: 245-252 (1998)
2. Bousso P., T-cell activation by dendritic cells in the lymph node: lessons from the movies. NATURE Rev. Immunol. 8 (2008) 675-684.
3. Keller E.F., L.A. Segel. Model for chemotaxis. J. Theor. Biol. 30 (1971) 225-234.
4. Klenin K., H. Merlitz, J. Langowski. A Brownian Dynamics Program for the Simulation of Linear and Circular DNA and Other Wormlike Chain Polyelectrolytes. Biophys. J. 74 (1998) 780–788.
5. Press W. H., S.A. Teukolsky, W.T. Vetterling, Flannery. Numerical recipes : the art of scientific computing, third edition, Cambridge University Press, New York, 2007.
6. Textor J., A. Peixoto, S.E. Henrickson, M. Sinn, U. Von Andrian, J. Westermann. Defining the quantitative limits of intravital two-photon lymphocyte tracking. PNAS, 108 (2011) 12401-12406.
7. Uhlenbeck G. E., L. S. Ornstein. On the Theory of the Brownian Motion. *Phys. Rev.* 36 (1930) 823-841.
